# To "E. P. M."

**Published:** 1888-04-07

**Authors:** 


### TO "E. P. M."

O fair are the halls where gay Peritonitis
Makes love to Miss Asthma and courts the Catarrh;
Where sweet Influenza is wooed by Tritis,
And Psora joins Measles in "Beautiful Star."
How bright gleam the eyes of that flirt Erythema,
And lightly Lumbago whirls round in the dance;
Pleuritis is madly in love with Œdema,
And Herpes courts Ague with languishing glance.

—*H. G. C.*

Garrick Club, London, W.C., March 26th, 1888.

